# Surface Analysis of Fermented Wheat and Rice Starch Used for Coating Traditional Korean Textiles

**DOI:** 10.3390/ma15062001

**Published:** 2022-03-08

**Authors:** Hye Hyun Yu, Youngseo Lee, Yun-Sik Nam, Man-Ho Kim, Kang-Bong Lee, Yeonhee Lee

**Affiliations:** 1 Advanced Analysis Center, Korea Institute of Science and Technology, Seoul 02792, Korea; 091651@kist.re.kr (H.H.Y.); gksten@kist.re.kr (Y.L.); ysnam@kist.re.kr (Y.-S.N.); man-hokim@kist.re.kr (M.-H.K.); 2 Center for Environment and Welfare Research, Korea Institute of Science and Technology, Seoul 02792, Korea; leekb@kist.re.kr

**Keywords:** starch, wheat, rice, fermented, ToF-SIMS, SEM

## Abstract

Wheat and rice starches, traditionally used to stiffen fabric, become less contaminated and more antiseptic after fermentation for several years, thus enhancing their functional activity. In the present study, analytical techniques using particle size analysis, a gloss meter and a colorimeter were used to measure the physical properties of wheat and rice starches that had been fermented for 5 and 7 years, respectively. Their chemical contents and composition were determined by nutrient measurements and time-of-flight secondary ion mass spectrometry (ToF-SIMS). The ToF-SIMS spectra and ion images showed that fermented starch contained more carbohydrate and less lipid than fresh starch. The surface morphologies of the fermented starch granules and starch-coated textiles were examined by scanning electron microscopy and compared with the surface morphologies of fresh starch granules. The fermented wheat and rice starch granules were smaller and more rounded with a lower level of N-containing compounds (proteins) and exhibited more antiseptic properties than fresh starch granules. The results showed that physical measurements and chemical analysis were simple and complementary techniques for investigating traditional Korean starch materials and textiles.

## 1. Introduction

Starch, a granular, organic chemical produced by green plants, is a white, tasteless powder that is insoluble in cold water, alcohol, or other solvents. Starch from several botanical materials is a polymeric carbohydrate containing mainly two types of polysaccharides, amylose and amylopectin [1,2,3,4,5,6,7]. The linear structure of amylose consists of glucose units joined by α-(1,4)-glycosidic linkages which are mainly catalyzed and elongated by granule-bound starch synthase. Amylopectin mainly consists of long chains of α-(1,4)-linked D-glucopyranosyl units with occasional branching α-(1,6)-linkages that form its branched structure. Starch usually comprises 20–25 wt.% amylose and 70–80 wt.% amylopectin depending on the plant source. Starch also contains small quantities of lipids, proteins and contaminants as minor components. Starch is the most common food in the human diet and is used commercially to produce sugars, alcohols and processed foods. The non-food use of starch for additives and papermaking is another major industrial application [8,9]. 

Many research groups have a lot of interest in coating materials and traditional textiles. Xia’s group had investigated Kemao knitted tapestry as well as Zuoqun made by the folk women in the Sudong region and H. Wang, et al. had provided useful information on other traditional textile fabrics and clothing technology [10,11,12]. Starch solutions have long been applied to stiffen textiles in Korea as well as other countries. They are prepared by mixing vegetable starch with water followed by boiling for a short time. During the Chosun Dynasty (1392–1910), laundry starch was widely used to stiffen fabrics, especially ramie because it has a cooling effect on the body when worn in the summer and could also style the clothes and protect the textiles. Starch fermented for several years was found to be more effective as a laundry starch because it improved the physical properties of the textiles, their smoothness, gloss, whiteness and comfort, and also provided an antiseptic effect. The surface morphology of starch has been investigated by several analytical techniques [13,14,15]. High-resolution images of wheat and potato starch granules can be obtained by low-voltage scanning electron microscopy (SEM) and atomic force microscopy (AFM) [13]. Images of rice starch granules have also been obtained by microscopy and confocal laser scanning microscopy before and after extracting proteins from the granule [15]. The nano-scale structural features of granules can also be observed by AFM. Recently, many starch science studies have used small-angle scattering techniques to quantify the lamellar architecture formed by side chains of amylopectin interspersed with amylose [16,17].

Surface analytical techniques can provide valuable information on the chemical composition of the surfaces, which is highly important for understanding the function of natural products and developing new materials [16,18,19,20,21,22]. X-ray photoelectron spectroscopy (XPS) has been used extensively to study wheat starch granules after various surface treatments [23]. ToF-SIMS has also been used to investigate the surface chemistry and composition of five different starch samples and identify specific ions from the starch granules [24].

However, the specific characterization of the surface of fermented starches used traditionally for laundry, adhesives and paper making has not yet been thoroughly studied. There is little information on the physical properties and chemical composition of the fermented starches although they have been used as clothing starch for a long time. The present study aims to characterize the surface of five-year fermented wheat starch and seven-year fermented rice starch and to compare the results with those of fresh wheat and rice starches using SEM and ToF-SIMS. The physical properties, granule size distribution, glossiness, color and antiseptic properties, will also be measured to determine if fermented starches provide any advantages over fresh starches for coating textiles. Thus, we obtained a profound understanding of traditionally fermented starch for several years in Korea.

## 2. Materials and Methods

### 2.1. Materials

The fresh and 5-year fermented wheat starch, and fresh and 7-year fermented rice starch were provided by Sunja Park, a craftswoman with great knowledge of traditional Korean clothes. The fermented starches had been prepared by milling the rice and wheat into a powder followed by fermentation in water for 5 or 7 years, respectively, using the traditional Korean method.

Two kinds of refined (uncoated) and starched textiles were also provided by Sunja Park, ramie and cotton fabrics. These are known as refined fabrics and were starched with glue made by boiling four types of starch. The fabrics were rinsed with starch glues, dried at room temperature to provide four finished products as starched textiles. For the SEM and ToF-SIMS measurements, the starch suspensions were prepared using 20 mg of starch in 1 mL distilled water then 2–5 μL of the starch solution were deposited onto a silicon substrate that had been thoroughly cleaned using methanol.

### 2.2. Methods

The particle size distributions of the starches were measured using a Zetasizer Nano-ZS ZEN 3600 (Malvern Panalytical, Malvern, UK) with a ZeN0040 cuvette at room temperature. The starch was dispersed in a solvent of dimethyl sulfoxide and dimethylformamide at a ratio of 3:1.

Glossiness is one of the most important factors for fabrics used in clothes making and varies with the type of fabric used and the extent of starching. The glossiness was measured using a Novo-Gloss Trio Glossmeter (Rhopoint Instruments Ltd., St. Leonards-on-Sea, UK) following the ASTM D523 standard.

The color of the starched fabrics was measured using a spectrum colorimeter (CM-3700A, Konica Minolta Inc., Osaka, Japan). A colorimeter measures color by illuminating a surface with white light then measuring the reflected light and assigning numerical values for L (brightness), a (redness and its complementary color, green as a negative value) and b (yellowness and its complementary color, blue as a negative value).

To compare the antibiotic effects of the fresh and fermented starches, the starch powders were mixed with cell culture media then observed for 24 h with *Escherichia coli* (ATCC, Manassas, VA, USA).

The nutritional contents of the starches were analyzed using methods recommended by the Korean Food Standards Codex from the Ministry of Food and Drug Safety [25]. The water contents were determined by drying the samples then heating them in an oven at 100 °C for 3–5 h. The amount of water was calculated as the reduction in weight after heating. The ash contents were determined by the reduction in weight of samples heated in an oven at 700 °C until the samples had become white. The fat contents were determined by the Soxhlet extraction method using circulating ether as the solvent. The contents of protein were determined by the semimicro-Kjeldahl method by calculating the total nitrogen then applying a conversion factor (6.25) to give the percentage of protein [26]. The samples were boiled in H_2_SO_4_ with a catalyst then the starch was added.

### 2.3. Instrumentation

The positive and negative ion ToF-SIMS analyses were made using a TOF-SIMS 5 system (IONTOF GmbH, Münster, Germany) with a base pressure of 3.0 × 10^−8^ Pa. The surface spectra and 2D ion images were measured in the high-current bunched mode with a pulsed 30-keV Bi_3_^+^ beam, which impacted the sample surface at an incident angle of 45° to the normal line. The pulsed current on the target was kept at 0.6 pA. A cycle time of 100 μs was used for the ion doses, well below the static SIMS limit of 10^13^ ions/cm^2^. Both the positive and negative secondary ions were accelerated from the sample surface by an ion lens operating at 2 kV into a time-of-flight (ToF) mass analyzer. The ToF used a reflector to decrease the energy distribution and line widths and had a total flight length of 2 m. The secondary ion beam was focused on the microchannel plate with a post-acceleration energy of 20 kV. A low-energy electron flood gun was used to compensate for the charge of the starch and textile samples. The mass spectra from the pure starch were collected from an area of 50 × 50 μm^2^ and used to determine the characteristic ions from the different starch samples. The spectra and 2D ion images were summarized using SurfaceLab 6.8 software (2021, IONTOF GmbH, Münster, Germany). The ToF-SIMS spectra were obtained from different areas of the starch granules by three measurements to confirm the ToF-SIMS characteristic ions and to obtain typical spectra.

The starch particles dispersed with water and coated on the Si wafer were observed using Field Emission Scanning Electron Microscopy (FE-SEM) to investigate the morphology and size of the starch particles. The observations were made at a voltage of 5 kV using an Inspect F50 instrument (FEI, Hillsboro, OR, USA) after the starch granules had been coated with Pt.

Attenuated total reflectance Fourier transform infrared spectroscopy (ATR-FTIR) spectra were obtained using a Nicolet iS20 FT-IR spectrometer (Thermo Fisher Scientific, Waltham, MA, USA) with a deuterated triglycine sulfate detector. The FTIR instrument uses a germanium ATR crystal which gave a shallow penetration depth. A spectrum was recorded over three times to confirm the measurement of each sample within the wavenumber region of 650–4000 cm^−1^ and 32 scans were collected with a resolution of 4 cm^−1^.

## 3. Results and Discussion

### 3.1. Properties of the Starch and Coated Textile Samples

**Figure 1:**
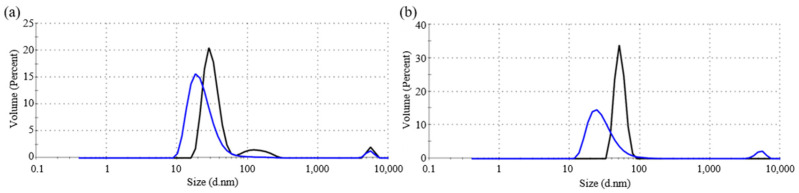
Size distribution by volume of: (**a**) fresh wheat starch and 5-year fermented wheat starch; and (**b**) fresh rice starch and 7-year fermented rice starch.

The size distributions of the fresh and fermented starch granules were obtained by dynamic light scattering (DLS) analysis as shown in Figure 1. The size of the fresh rice starch granules was greater than that of the wheat starch granules and became smaller after fermentation. Table 1 lists the size and percentage volume of the starch granules before and after fermentation. The DLS results revealed that the size distribution of the starch granules decreased after long-time fermentation.

The compositions of the different starch granules were measured (Table 2). The fresh wheat starch contained more protein (10.2%) and fat (1.33%) than the fresh rice starch. The amount of carbohydrates in the fermented starch increased but the amount of protein decreased after the starch granules had been fermented.

**Figure 2:**
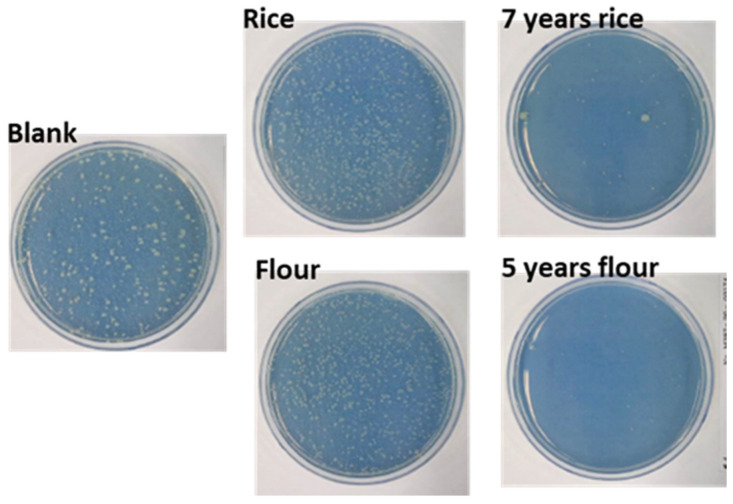
Antibiosis experiments on fresh wheat starch, 5-year fermented wheat starch, fresh rice starch and 7-year fermented rice starch.

In Figure 2, cell culture experiments were used to evaluate the antibiotic properties of the fresh and fermented starches. Table 3 shows the changes in the numbers of Escherichia coli. cultivated in four different experimental environments: fresh wheat, 5-year fermented wheat, fresh rice and 7-year fermented rice starches. The number of cells in each Petri dish was counted after 24 h and then compared with that of the control blank sample. The cells grown on fermented starch had almost completely disappeared after 24 h, decreasing by 98.8% and 99.9% for 5-year fermented wheat and 7-year fermented rice starches, respectively. These results indicated that fermented wheat and rice starch exhibited excellent antiseptic properties compared with fresh starch.

The ramie and cotton fabrics were coated with fresh and fermented starch (wheat and rice) then their appearance was measured using a glossmeter and colorimeter. Table 4 shows that the glossiness and brightness of textiles coated with starch exhibited higher values compared with those of the uncoated refined fabrics. The ramie textiles coated with fermented starch showed glossier and brighter values than those with fresh starches. There were no significant differences in glossiness and color between cotton textiles coated with fresh and fermented starches.

### 3.2. SEM Images of the Starch and Textile Samples

**Figure 3:**
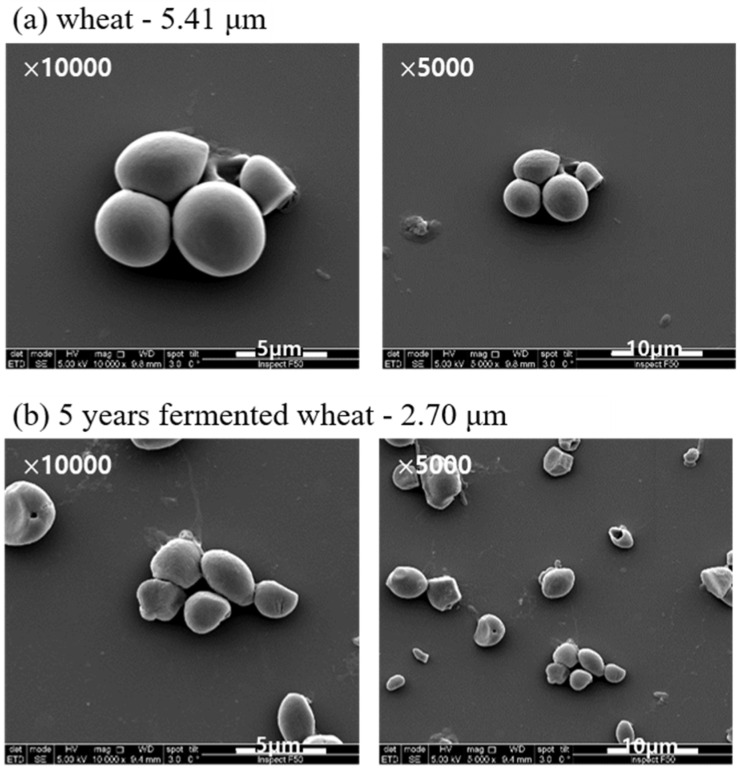

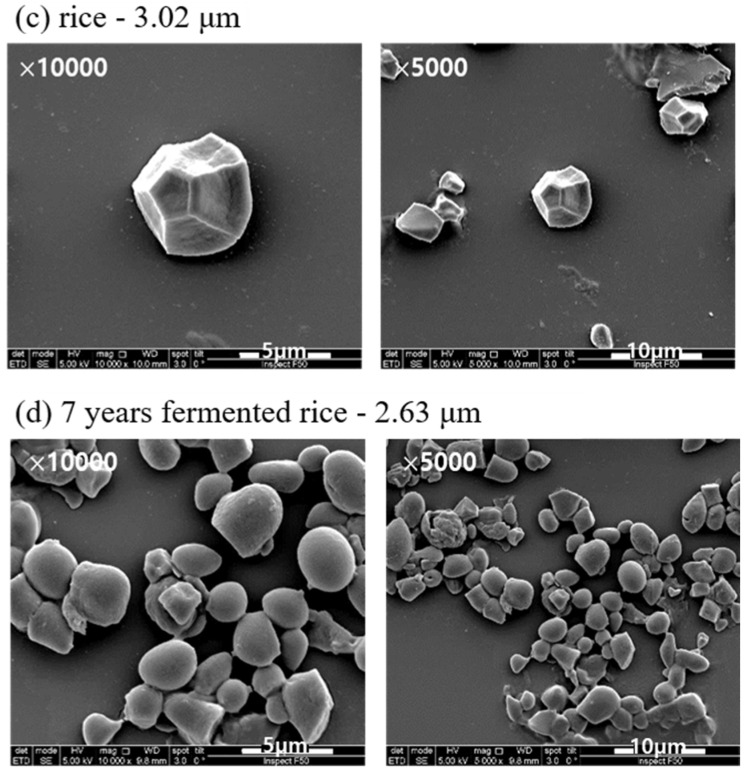
SEM micrographs of: (**a**) fresh wheat starch; (**b**) 5-year fermented wheat starch; (**c**) fresh rice starch; and (**d**) 7-year fermented rice starch granules.

The effect on the morphology of the different types of starch granules is shown in Figure 3 in the SEM images of fresh wheat starch, 5-year fermented wheat starch, fresh rice starch and 7-year fermented rice starch granules. The shape of the starch granules differed: the fresh wheat starch granules were very round with a smooth outline with some attached to each other to form larger granules. In contrast, the rice starch granules exhibited a polyhedral and edged shape. The SEM images of fresh rice starch granules are well agreed with other research results [15]. The images of some granules were made at a higher magnification to show the detailed surface morphology. The SEM images of wheat and rice starch showed smaller granules after fermenting the starch for several years than those of the fresh starch. The fermented rice starch granules exhibited a worn structure having lost their original angular outline.

**Figure 4:**
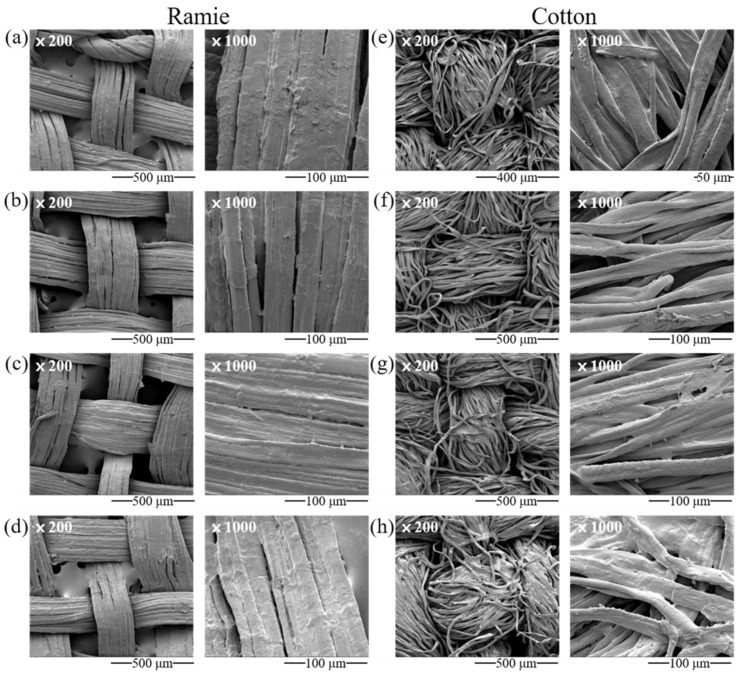
SEM micrographs of ramie and cotton fabrics coated with: (**a**,**e**) fresh wheat starch; (**b**,**f**) 5-year fermented wheat starch; (**c**,**g**) fresh rice starch; and (**d**,**h**) 7-year fermented rice.

Starch used for clothing is a solution prepared by mixing and boiling the starch powder in water into which the ramie and cotton fabrics were immersed. Figure 4 shows the SEM images of ramie and cotton fabrics coated with fresh and fermented starch. The SEM images for the ramie fabrics coated with fermented wheat and rice starch showed a more uniform and smoother coated surface than coated with fresh starch. This result can be expected from the properties of the fermented granules that were smaller in size, more uniform, and less viscous than fresh granules.

### 3.3. ToF-SIMS Analyses of the Starch and Textile Samples

**Figure 5:**
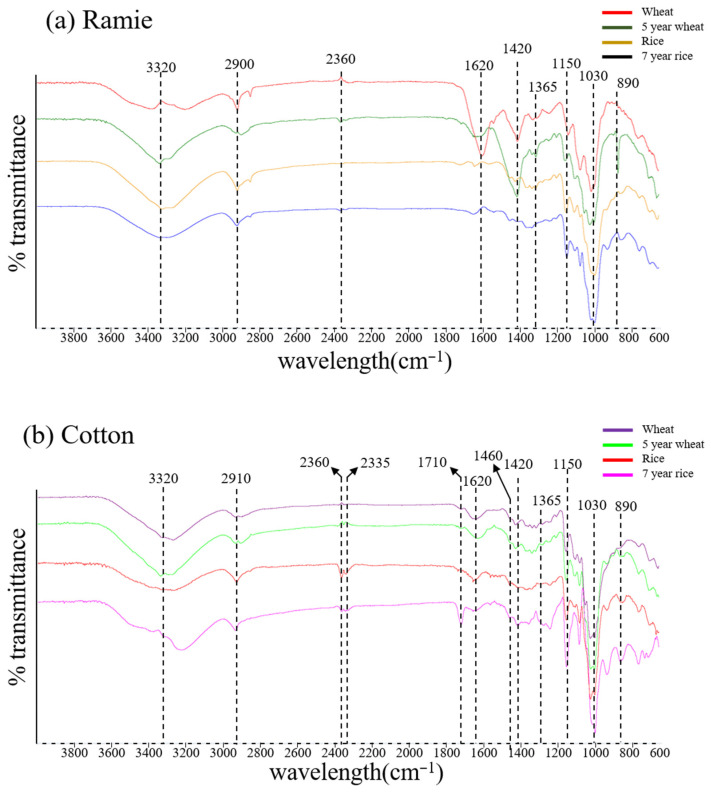
FT-IR spectrums of (**a**) Ramie (**b**) Cotton fabrics coated with fresh wheat starch, 5-year fermented wheat starch, fresh rice starch, and 7-year fermented rice.

Figure 5 compares the ATR FT-IR spectra of ramie and cotton fabrics coated with fresh and fermented wheat and rice starch. Uncoated ramie and cotton fabrics were used to obtain the reference spectrum. The peaks at 3320 cm^−1^ are due to O-H stretching vibration, and the peaks at 2910 and 2855 cm^−1^ are due to the C-H stretching and the =C-H stretching vibrations. The peak at 1460 cm^−1^ is due to C-H bending in a side chain and the peaks at 1150 cm^−1^ and 1030 cm^−1^ indicate C-O stretching vibration. The spectrum of textiles with wheat and rice starch is shown with a specific peak at 2360 cm^−1^ due to the N-H stretching, a peak at 1620 cm^−1^ that is because of C=O stretching. These results, which are expected because starch is the main coating component support the accuracy of the FT-IR technique. However, it was not possible to distinguish fresh starch and fermented starch in textiles using the ATR FT-IR technique.

Information on the molecular structure and chemical composition of the top surface of the materials was obtained by the ToF-SIMS technique. The negative ion ToF-SIMS mass spectra of the starch granules were measured. Figure 5a shows the specific fragment ion peaks from the fresh wheat starch and 5-year fermented wheat starch granules. The typical spectrum of fresh wheat starch granules in the low mass range (*m*/*z* 1–100) showed the intense peaks of CN^−^ (*m*/*z* 26.00), CNO^−^ (*m*/*z* 41.99), C_2_H_3_O_2_^−^ (*m*/*z* 59.01), C_3_H_3_O_2_^−^ (*m*/*z* 71.01), C_3_H_3_O_3_^−^ (*m*/*z* 87.01) and C_4_H_3_O_3_^−^ (*m*/*z* 99.01). In the higher mass range (*m*/*z* 100–360), the major peaks of carbohydrate molecules occurred at *m*/*z* 179 (C_6_H_11_O_6_^−^) and *m*/*z* 221 (C_8_H_13_O_7_^−^). Other intense peaks at *m*/*z* 255, 279, 311, 325 and 339 can be assigned to lipid molecules as well as carbohydrate fragment ions. The characteristic peaks at *m*/*z* 255, 279 and 339 could have arisen from free fatty acids, such as palmitic, linoleic and behenic acids. The peaks at *m*/*z* 311 and 325 were indicative of the presence of arachidic acid ion fragments, generated from free fatty acids and/or from glycerides in the starch granules. The ToF-SIMS spectra of the fresh starch granules provided identification of specific ions generated from granule surface carbohydrates, lipids, and contaminants that were consistent with the results of Baldwin’s group [24].

**Figure 6:**
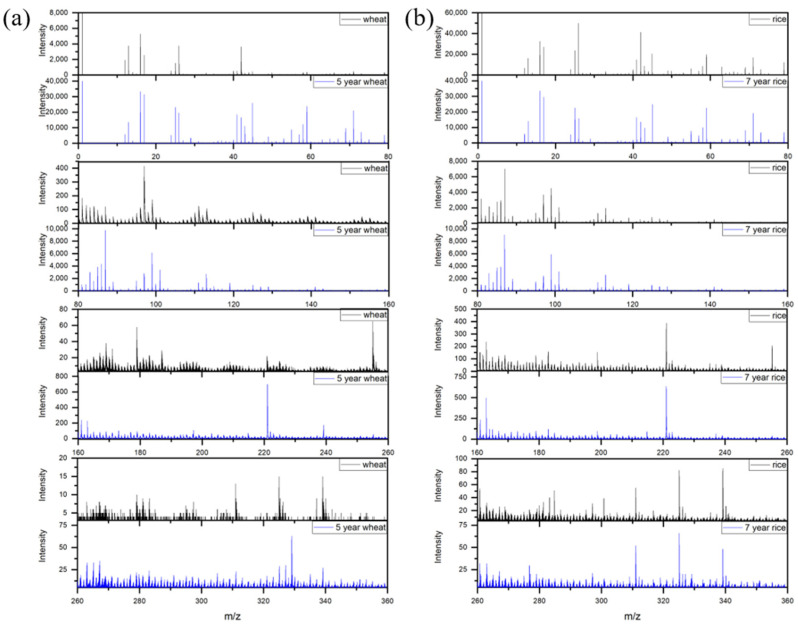
The negative ion ToF-SIMS spectra of: (**a**) fresh wheat starch and 5-year fermented wheat starch; and (**b**) rice starch and 7-year fermented rice starch in the mass range, *m*/*z* 0–360.

Figure 6a shows the ToF-SIMS spectra of 5-year fermented wheat starch compared with those of fresh wheat starch. The fragment ion intensities at *m*/*z* 87, 99 and 221 from the carbohydrate molecules of the 5-year fermented wheat starch increased but the characteristic peaks from carbohydrate molecular ions and free fatty acids significantly decreased. Figure 6b also compares the ToF-SIMS spectra of the fresh rice and 7-year fermented rice starch granules. Some characteristic peaks from the rice starch granules were observed and found to be similar after fermentation. The positive ion spectra of the fresh and fermented starches exhibited similar peaks from carbohydrates and lipids (not shown).

**Figure 7:**
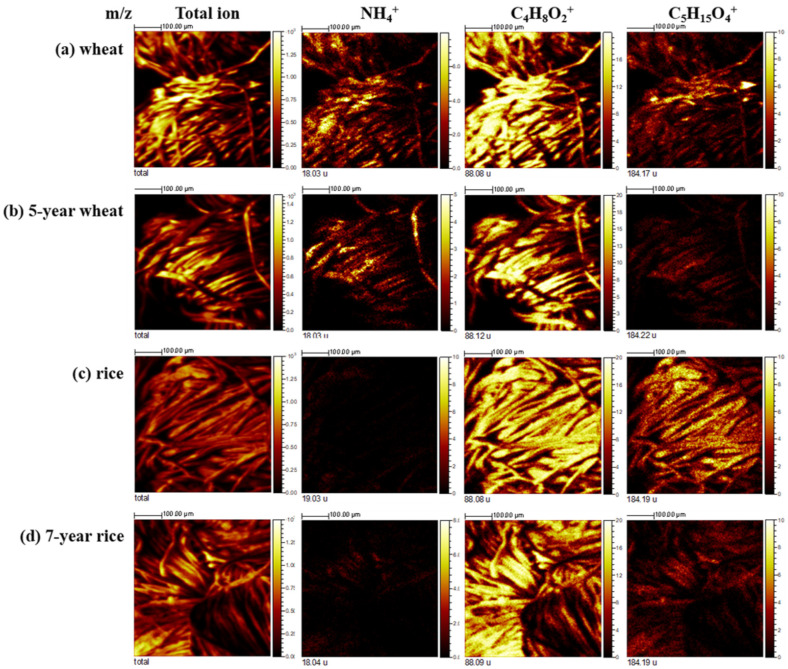
The positive ion ToF-SIMS images of total ion, *m*/*z* 18, 88 and 184 for cotton textiles coated with: (**a**) fresh wheat starch, (**b**) 5-year fermented wheat starch; (**c**) fresh rice starch, and (**d**) 7-year fermented rice starch.

The textile samples were coated with the four different starch solutions prepared from fresh wheat, 5-year fermented wheat, fresh rice and 7-year fermented rice starches. The negative ion ToF-SIMS spectra were obtained from these four textile samples. The characteristic peaks from the carbohydrate molecules and lipid molecules were easily observed in the ToF-SIMS spectra of the starch-coated textiles. The ToF-SIMS measurements enabled the chemical distribution of the starch component on the cotton fabric to be easily evaluated. The ToF-SIMS imaging experiment was performed on cotton fabric samples coated with fresh and fermented wheat and rice starch. Figure 7, showing the ion images of the cotton fabric samples measured using the positive ion ToF-SIMS technique, revealed characteristic ions of NH_4_^+^, C_4_H_8_O_2_^+^ and C_5_H_15_O_4_NP^+^ as well as total ions. The local distribution of the molecular fragments of carbohydrates and fatty acids (lipids) was homogeneous as shown over the images. The more intense peaks of phospholipids and free fatty acids were present in the ion images of cotton textiles coated with fresh starch. The ToF-SIMS images of textile samples coated with fresh wheat starch exhibited more N-containing ions than those coated with fresh rice starch expected from the composition of the fresh wheat starch. The ToF-SIMS image results confirmed that cotton textiles coated with fermented wheat and rice starch had fewer lipid and N-containing components, such as proteins compared with those coated with fresh starch.

**Figure 8:**
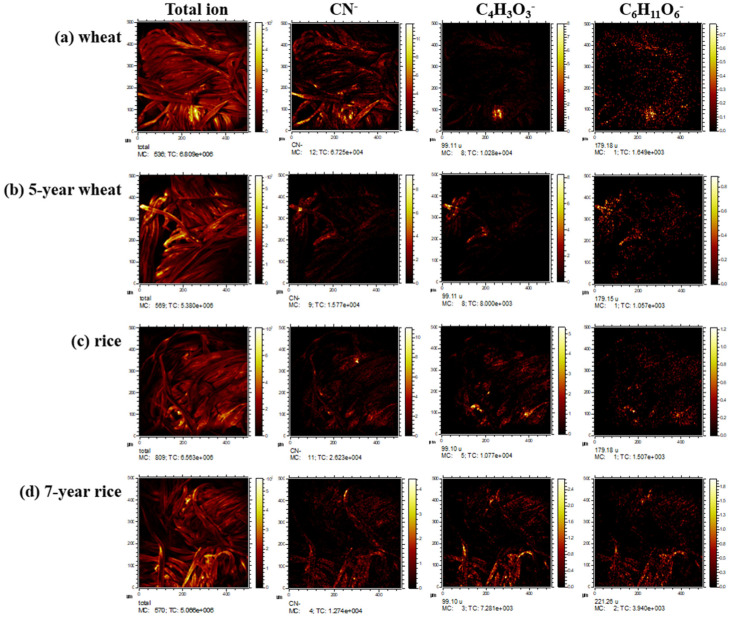
The negative ion ToF-SIMS images of total ion, *m*/*z* 26, 99 and 179, for cotton textiles coated with: (**a**) wheat starch; (**b**) 5-year fermented wheat starch; (**c**) rice starch and (**d**) 7-year fermented rice starch.

As shown in Figure 8, the negative ion ToF-SIMS images were also obtained of cotton textiles coated with fresh wheat starch, 5-year fermented wheat starch, fresh rice starch, and 7-year fermented rice starch. An intense specific peak, CN^−^ from proteins and lipids, was observed in textiles coated with fresh wheat starch. The molecular ions of carbohydrates and fatty acids, such as C_4_H_3_O_3_^−^ and C_6_H_11_O_6_^−^ appeared to be located in small aggregates. The ToF-SIMS technique has provided useful information on both the elements, including isotopic distribution and the molecules present on textiles coated with fresh and fermented starch.

## 4. Conclusions

Complementary chemical and physical analysis methods have been used to gain a better understanding of the use of traditionally fermented starch granules for coating textiles. Many research groups have studied starch and textiles, however, research about starch fermented for several years is barely found. Wheat and rice starch granules were fermented for 5 and 7 years, respectively, using a traditional process. The fermented starch granules and their coated textiles were examined by physical and structural methods using a gloss meter, colorimeter, particle size analysis and SEM. The fermented starch granules exhibited a smaller particle size and smoother shape than the fresh starch granules and the glossiness and brightness of ramie textiles coated with fermented starch were better than those coated with fresh starch. The SEM images indicated that the rice starch granules had a very characteristic shape before fermentation which became round and small after fermentation. The antiseptic properties of the fermented starches were highly improved compared with those of the fresh starches. The surface technique, ToF-SIMS, allowed the acquisition of useful information on both the fresh and fermented starch granules and the textiles coated with traditionally fermented starch. The important benefits of using ToF-SIMS to characterize starch materials include the minimal sample size, the lack of sample preparation and the identification of the elemental and organic components of the starch materials. Establishing physical and chemical methods using a systematic approach will provide a deeper understanding of traditional techniques and wisdom for treating fabric materials. Thus, establishing physical and chemical methods using a systematic approach provide a deeper understanding of traditionally fermented starches and coated textiles.

## Figures and Tables

**Table 1:** Size and percentage volume of four different types of starch particle and amylose and amylopectin.

		Peak 1	Peak 2	Peak 3
Wheat	Size ± SD (d.nm) % Volume	31.1 ± 8.2 85.6	139.1 ± 49.2 10.4	5590.0 ± 579.8 4
5-year fermented wheat	Size ± SD (d.nm) % Volume	24.6 ± 18.2 97	5433.0 ± 643.0 3	0 0
Rice	Size ± SD (d.nm) % Volume	52.3 ± 8.7 100	0 0	0 0
7-year fermented rice	Size ± SD (d.nm) % Volume	30.9 ± 19.5 93.8	5159.0 ± 773.3 6.2	0 0
Amylose	Size ± SD (d.nm) % Volume	8.9 ± 2.2 64.6	29.7 ± 12.5 32.6	401.6 ± 147.6 2.8
Amylopectin	Size ± SD (d.nm) % Volume	54.8 ± 15.6 28	401.5 ± 140.0 31.4	5385.0 ± 668.6 40.6

**Table 2:** Nutritional composition of fresh wheat starch, 5-year fermented wheat starch, fresh rice starch and 7-year fermented rice starch.

	Carbohydrate %	Protein %	Fat %	Water %	Ash %
Wheat	78.06	10.21	1.33	10.25	0.15
5-year fermented wheat	86.74	0.41	0.78	11.98	0.09
Rice	81.14	6.37	0.79	11.62	0.08
7-year fermented rice	86.89	0.49	0.86	11.74	0.02

**Table 3:** Antiseptic properties of fresh wheat starch, 5-year fermented wheat starch, fresh rice starch and 7-year fermented rice starch.

	Blank	Wheat	5-Year Wheat	Rice	7-Year Rice
Initial (CFU/mL)	2.1 × 10^5^	2.1 × 10^5^	2.1 × 10^5^	2.1 × 10^5^	2.1 × 10^5^
24 h (CFU/mL)	1.3 × 10^5^	4.8 × 10^5^	1.6 × 10^3^	4.8 × 10^5^	1.8 × 10^2^
Percentage reduction	-	0	98.8%	0	99.9%

**Table 4:** Glossiness and brightness of fresh wheat starch, 5-year fermented wheat starch, fresh rice starch and 7-year fermented rice starch.

	Ramie				Cotton			
	Glossiness Units(GU)	L* (Brightness)	a* (Red)	b* (Yellow)	Glossiness Units(GU)	L* (Brightness)	a* (Red)	b* (Yellow)
Refined	2.50	65.4	−0.52	1.79	2.28	81.3	0.98	1.62
Wheat	2.61	68.4	−0.52	1.28	2.60	81.2	0.89	1.74
5-year fermented wheat	2.72	68.2	−0.27	3.6	2.55	79.3	0.9	1.5
Rice	2.60	64.6	−0.43	1.62	2.50	81.8	1.06	1.48
7-year fermented rice	2.62	68.0	−0.5	1.54	2.51	81.0	1.07	1.9

L* is the degree of white and black, a* is the degree of red and green, and b* is the degree of yellow and blue.

## Data Availability

Data are contained within the article.
